# Susceptibility to prosocial and antisocial influence in adolescence

**DOI:** 10.1016/j.adolescence.2020.07.012

**Published:** 2020-10

**Authors:** S. Ahmed, L. Foulkes, J.T. Leung, C. Griffin, A. Sakhardande, M. Bennett, D.L. Dunning, K. Griffiths, J. Parker, W. Kuyken, J.M.G. Williams, T. Dalgleish, S.J. Blakemore

**Affiliations:** a Institute of Cognitive Neuroscience, University College London, London, WC1N 3AR, UK; b Medical Research Council Cognition and Brain Sciences Unit, Cambridge University, Cambridge, CB2 7EF, UK; c Department of Psychiatry, University of Oxford, Oxford, OX3 7JX, UK; d Department of Psychology, Downing Street, University of Cambridge, Cambridge, CB2 3EB, UK

**Keywords:** Adolescence, Social influence, Prosocial, Antisocial, Puberty, Social cognitive development

## Abstract

**Introduction:**

Adolescents are particularly susceptible to social influence and previous studies have shown that this susceptibility decreases with age. The current study used a cross-sectional experimental paradigm to investigate the effect of age and puberty on susceptibility to both prosocial and antisocial influence.

**Methods:**

Participants (N = 520) aged 11–18 from London and Cambridge (United Kingdom) rated how likely they would be to engage in a prosocial (e.g. “help a classmate with their work”) or antisocial (e.g. “make fun of a classmate”) act. They were then shown the average rating (in fact fictitious) that other adolescents had given to the same question, and were then asked to rate the same behaviour again.

**Results:**

Both prosocial and antisocial influence decreased linearly with age, with younger adolescents being more socially influenced when other adolescents’ ratings were more prosocial and less antisocial than their own initial rating. Both antisocial and prosocial influence significantly decreased across puberty for boys but not girls (independent of age).

**Conclusions:**

These findings suggest that social influence declines with increasing maturity across adolescence. However, the exact relationship between social influence and maturity is dependent on the nature of the social influence and gender. Understanding when adolescents are most susceptible to different types of social influence, and how this might influence their social behaviour, has important implications for understanding adolescent social development.

## 1 Introduction

Individuals tend to adopt the opinions, judgements, and behaviour of other people in order to fit in with them (Turner, 1991; Zaki, Schirmer, & Mitchell, 2011). The degree of conformity is age-dependent, with studies showing that susceptibility to social influence is highest during late childhood and adolescence and then steadily declines into adulthood (Foulkes, Leung, Fuhrmann, Knoll, & Blakemore, 2018; Knoll, Leung, Foulkes, & Blakemore, 2017; Knoll, Magis-Weinberg, Speekenbrink, & Blakemore, 2015; Steinberg & Monahan, 2007; Sumter, Bokhorst, Steinberg, & Westenberg, 2009).

Adolescence is defined as the period between the onset of puberty and the achievement of a stable adult role in society, approximately 10–24 years (Sawyer, Azzopardi, Wickremarathne, & Patton, 2018). Significant social re-orientation takes place during adolescence as more time is spent with peers than with family (Lam, McHale, & Crouter, 2014; Larson & Richards, 1991; Nelson, Leibenluft, McClure, & Pine, 2005; van den Bos, 2013), and peers become an additional important source of social influence (De Goede, Branje, Delsing, & Meeus, 2009). Several studies have shown that adolescents are particularly sensitive to peer rejection (Peake, Dishion, Stormshak, Moore, & Pfeifer, 2013; Sebastian et al., 2011; Somerville, 2013) and social approval (Foulkes & Blakemore, 2016). The heightened susceptibility to social influence in adolescents, combined with the increased fear of social rejection, increases the likelihood that adolescents will conform to their peers in order to gain social acceptance (Blakemore & Mills, 2014).

### 1.1 Social influence in adolescence

Social influence is often a prosocial process; individuals of all ages can be influenced by others to behave in a way that benefits other people or society such as cooperation, donation, and volunteering (Barry & Wentzel, 2006; Padilla-Walker & Carlo, 2014; van Hoorn, van Dijk, Meuwese, Rieffe, & Crone, 2016). Several studies have investigated such prosocial influence across the lifespan. In adults, learning about other people's prosocial actions has been associated with donating more generously to charity (Frey & Meier, 2004; Nook, Ong, Morelli, Mitchell, & Zaki, 2016; Shang & Croson, 2009), acting more fairly in economic games (Fowler & Christakis, 2010; Peysakhovich & Rand, 2015), and protecting the environment (Goldstein, Cialdini, & Griskevicius, 2008). A similar pattern has been seen in adolescence, with participants distributing coins more generously to their group after they observed peers approve such behaviour (van Hoorn et al., 2016) and being more likely to volunteer to help others in their community if they believed other students in their school, particularly high-status students, were already volunteering (Choukas-Bradley, Giletta, Cohen, & Prinstein, 2015).

Foulkes et al. (2018) investigated differences in susceptibility to prosocial influence between childhood and adulthood. Unlike the studies described above, social influence effects here pertained to changes in hypothetical actions and not actual behaviours. Participants were first asked to rate how likely they would be to engage in a prosocial behaviour, such as ‘Give up your seat on the bus’. They were then shown the average rating provided by (fictitious) previous participants, and were finally asked to re-rate the same scenario for themselves. The study found that children (8–11 years), young adolescents (12–14 years) and mid-adolescents (15–18 years) all significantly changed their ratings in line with the feedback, while young adults (19–25 years) and adults (26–59 years) did not.

Individuals can also influence each other to behave more antisocially, i.e. in ways that are potentially harmful to other people. Antisocial influence is common at all ages (Monahan, Steinberg, & Cauffman, 2009; Slattery & Meyers, 2014), but tends to peak during adolescence, with several studies demonstrating that peer influence is an important contributor to many types of antisocial and risky behaviours in adolescence. This includes minor delinquency, serious offending, reckless driving and bullying (Doehne, von Grundherr, & Schäfer, 2018; Espelage, Holt, & Henkel, 2003; Shope, Raghunathan, & Patil, 2003; Simons-Morton, Lerner, & Singer, 2005). In experimental studies, the number of risks taken by adolescents during a simulated driving game increased almost threefold when they were being watched by friends compared to when alone, whereas this was not the case for adults (Chein, Albert, O'Brien, Uckert, & Steinberg, 2011; Gardner & Steinberg, 2005).

Antisocial influence also occurs for indirect forms of antisocial behaviour such as gossiping and ostracising others. This type of antisocial behaviour has been associated with achieving a high social status; adolescents are more likely to be influenced by peers who engage in this type of behaviour as those peers are often popular (Card, Stucky, Sawalani, & Little, 2008; Garandeau & Cillessen, 2006; Heilbron & Prinstein, 2008). For example, 11-year-olds were asked to nominate which of their classmates were bullies and which of their classmates they thought were “cool” (Juvonen & Ho, 2008). Children who perceived bullies as also being cool were likely to show increases in their own bullying behaviour a year later (Juvonen & Ho, 2008). Furthermore, using longitudinal social network analysis on a sample on children (aged 9–10) and young adolescents (aged 11–14), Sijtsema, Rambaran, Caravita, and Gini (2014) found that young adolescents, but not children, selected peers as friends who were similar in levels of bullying perpetration (ostracising others), and became more similar to friends in this behaviour one year later. The findings suggest that young adolescents are more susceptible to being influenced by indirect antisocial behaviour than are children.

### 1.2 Puberty and social influence

The majority of studies to date have investigated the development of susceptibility to social influence across chronological age. However, research has shown that puberty also plays an important role in adolescent social outcomes (Waylen & Wolke, 2004) and social-affective development (Crone & Dahl, 2012). Puberty is the period during which adolescents reach sexual maturity and become capable of reproduction, which typically begins between 9 and 12 years of age (usually 1–2 years earlier in girls than in boys; Kail & Cavanaugh, 2010). Hormonal changes occurring during puberty have a direct effect on the adolescent brain, which in turn influences the individual's mental state and behaviour (Cameron, 2004; Dahl, 2004; Sisk & Foster, 2004). Given that there is normal variation of around five years in the timing of the onset of puberty (Parent et al., 2003), pubertal development is partially dissociable from chronological age. Several studies have shown that pubertal status—independent of chronological age—influences the structure and function of brain regions implicated in social cognition (Bramen et al., 2012; Forbes et al., 2010; Goddings, Burnett Heyes, Bird, Viner, & Blakemore, 2012; Wierenga et al., 2018). Therefore, when examining the development of social influence, as with any other social cognitive process, it may be informative to examine the effects of puberty as well as chronological age.

To date, no studies have directly assessed the impact of puberty on prosocial or antisocial influence, but several studies on related phenomena suggest that puberty may be relevant. First, adolescents aged 11–14 with more advanced pubertal development report higher levels of sensation-seeking and greater drug use, independent of age (Martin et al., 2002), which has been related to increased peer influence (Wang et al., 2016). Second, in a study of adolescents aged 12–17, an increase in negative self-evaluation was uniquely associated with pubertal maturation and not age (Ke, Wu, Willner, Brown, & Crowley, 2018). Since low self-esteem and fear of ostracism play a role in social influence (Carter-Sowell, Chen & Williams, 2008; Uslu, 2013), these findings suggest that puberty may also therefore independently affect susceptibility to social influence.

It is possible that there are gender (or sex) differences in the experience of puberty and susceptibility to social influence (Negriff, Ji, & Trickett, 2011). Studies have shown that advanced pubertal status is associated with increased reactivity to social rejection in brain regions implicated in social and affective processing, independent of age (Silk et al., 2014), and this increasing rejection sensitivity is found for girls but not boys across development (Stroud, Papandonatos, D'Angelo, Brush, & Lloyd-Richardson, 2017). Moreover, advanced pubertal status at age 11 years was associated with higher levels of social anxiety, only in girls (Deardorff et al., 2007). Both sensitivity to social rejection and social anxiety may be relevant for understanding susceptibility to social influence. Given that puberty influences social behaviour, the present study investigated how susceptibility to social influence varies with pubertal status in addition to chronological age in boys and girls separately.

### 1.3 The current study

The current study used a similar paradigm to Foulkes et al. (2018) in order to assess the effect of four variables: participant age, type of social information (*prosocial* or *antisocial*), direction of influence (whether other people report being more or less likely than you to engage in a behaviour; Knoll et al., 2017) and pubertal status, on susceptibility to social influence in a large group of participants aged 11–18 years. This age range was chosen as it is the period when social influence appears to undergo the most change (Chein et al., 2011; Foulkes et al., 2018; Gardner & Steinberg, 2005; Knoll et al., 2017; 2015), perhaps due to adolescents being hypersensitive to peer rejection (Peake et al., 2013; Sebastian et al., 2011; Somerville, 2013) and social approval (Foulkes & Blakemore, 2016). Although it has been well documented that peers play an important role in prosocial and antisocial influence during adolescence, these processes have not been investigated within participants in a single experimental task, and to date the effect of puberty has not been assessed.

Susceptibility to social influence was measured here as *the extent to which participants change reports of their own prosocial/antisocial behaviour after seeing how much others endorse the same prosocial/antisocial behaviour*. Participants first rated how likely they would be to engage in a prosocial/antisocial behaviour, such as carrying someone's bag for them or stealing someone's bag (Rating 1). Participants were then presented with the ‘average rating’ that other participants gave for the same behaviour (‘provided rating’; participants were informed that the rating is from other adolescents, but in fact provided ratings were randomly generated). Finally, participants re-rated how likely they would be to engage in the same behaviour (Rating 2). We had four hypotheses:

1) *Age differences in susceptibility to social influence:* The extent to which participants change their ratings from Rating 1 to Rating 2 for both prosocial and antisocial behaviour will decrease with age. This is based on previous evidence that the magnitude of susceptibility to social influence (for risky or antisocial behaviour and prosocial behaviour) decreases over age (Foulkes et al., 2018; Knoll et al., 2015; Steinberg & Monahan, 2007; Sumter et al., 2009).
2) *Effect of social condition:* The extent of social influence will be affected by the type of social condition (*prosocial* or *antisocial* actions). Given the lack of previous research comparing antisocial and prosocial influence in the same study, this hypothesis was non-directional.
3) *Direction of influence:* the extent of social influence will be affected by whether the provided rating is higher or lower than participant's Rating 1 and this would be different depending on the social condition.
4) *Puberty-related differences in susceptibility to social influence:* The extent to which participants change their ratings will be affected by pubertal status (controlling for age), but we had no strong directional hypotheses here.

## 2 Method

### 2.1 Participants

Participants were recruited as part of a large-scale UK study investigating the mechanisms, effectiveness, cost effectiveness and scalability of mindfulness training in adolescence. Data from 552 participants (before mindfulness training commenced) from 12 schools in Greater London and Cambridgeshire were collected alongside a range of other cognitive tasks and questionnaires. IQ was measured using Cattell's Culture Fair Intelligence test (Institute for Personality and Ability, 1973). Data from 32 participants were excluded from the analysis, either because the participant did not complete the social influence task (n = 21), they were not attending to the task (n = 4), they had an SEN requirement (n = 1) or they were missing IQ data (n = 6). In total, data from 520 participants aged 11.2–18.5 (M = 14.33, SD = 1.74; IQ ranged from 62 to 160; M = 111.4; SD = 17.14) were analysed for Hypotheses 1, 2 and 3. Of the 520 participants, only 369 participants had puberty data and were therefore included in the analysis for Hypothesis 4 (see *Puberty measure* below for more detail).

The study was approved by the UCL Research Ethics Committee. Informed consent from parents and assent from all participants was obtained. Participants were compensated £15 in vouchers for taking part in a 3-h testing session, which was held at the participants’ school. The majority of testing took place in groups (comprising between 2 and 15 participants); one participant was tested by themselves as they missed the testing session. Due to school scheduling constraints, testing sessions were split over two days for four groups of participants (N = 46). All other testing sessions were completed in one day.

### 2.2 Puberty measure

Pubertal status was measured using the Pubertal Development Scale (PDS; Petersen, Crockett, Richards, & Boxer, 1988), a self-report scale that assesses five general indicators of development (growth in height, skin changes, growth of body for both boys and girls; facial hair growth and voice change for boys only; and breast development and menarche for girls only). Responses are coded on 4-point scales (1 = no development and 4 = completed development). For girls, a question about onset of menarche was rated on a 3-point scale (1 = no and 3 = yes definitely). Respondents on the PDS can be grouped into puberty categories using several methods. Pubertal development is traditionally classified into five Tanner stages - prepubertal, early pubertal, mid-pubertal, late pubertal, and post-pubertal (Carskadon & Acebo, 1993). However, given the unbalanced number of participants across the five groups, particularly the lack of prepubertal and a small number of early pubertal participants (N = 49), we divided participants into two groups: early/mid (stages 2 & 3) and late/post puberty (stages 4 & 5) (Chan, Choi, Nelson, Chan, & Kong, 2015; Deardorff et al., 2007; see Table 1). Girls in the early/mid group were pre-menarche and girls in the late/post group were post-menarche (e.g. Burnett, Thompson, Bird, & Blakemore, 2011). Boys in the early/mid group had low individual ratings on growth of body hair, voice change, and growth of facial hair growth compared to boys in the late/post group (see Norris & Richter, 2008, for scoring details).

**Table 1:** Participants separated by pubertal status.

Pubertal status	Boys	Mean age (range)	Girls	Mean age (range)
**Early/mid**	99	13.31	64	12.80
		(12–16)		(12–18)
**Late/post**	31	15.48	175	14.93
		(13–18)		(11–18)
**Total**	**130**		**239**	

## 3 Social influence task

### 3.1 Stimuli

**Fig. 1:**
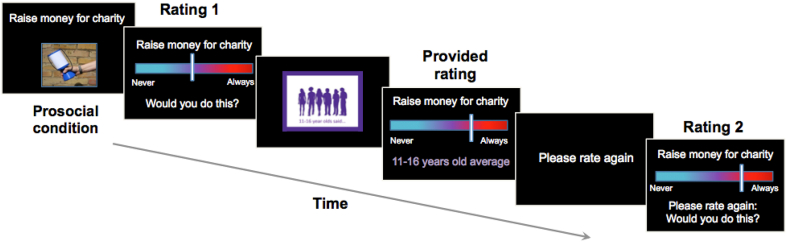
Trial sequence (prosocial trial shown). Participants were asked to rate how likely they would be to engage in the behaviour (Rating 1). They were then shown the average rating of other adolescents (provided rating) and asked to rate the scenario again (Rating 2).

Eighty-two scenarios (41 prosocial and 41 antisocial, see Supplementary Material for full list) were created for this task, each describing a social behaviour (e.g. prosocial: “Give money to charity”; antisocial: “Make fun of a classmate”). An image depicting the scenario was included to make the task more engaging (see Fig. 1). The list of prosocial behaviours was adapted from a previous task assessing prosocial influence (Foulkes et al., 2018), with adaptations made to ensure the behaviours were relevant to the current adolescent age group. Prosocial scenarios covered helping and sharing behaviours towards strangers, family, and friends; giving to charity; and prosocial risk behaviours such as defending classmates. The list of antisocial scenarios was devised for this study and covered a range of behaviours relevant to adolescents, including violation of privacy, indirect aggression (e.g. gossiping), direct aggression (verbal, physical), theft and vandalism. Following Foulkes et al. (2018), moderately prosocial and antisocial behaviours that could reasonably elicit a variety of response ratings were chosen, to ensure that the randomly generated provided rating (supposedly the average rating from other participants) would be believable across the full range of the scale. All scenarios were rated on clarity and age-appropriateness by five independent raters with expertise in adolescent social cognition. Scenarios that were kept had consensus on clarity and age appropriateness.

### 3.2 Procedure & trial sequence

Participants completed the task in groups at their school, guided by a team of three or four researchers. The laptops were sufficiently spread out so that participants could not see each other's screens or talk to anyone else while taking part. Before the task, participants read instructions on the screen and were shown an example trial. They then completed a practice trial and had the opportunity to ask any questions. Each participant completed 16 trials (eight for each social condition; prosocial and antisocial) randomly selected out of the possible 82 scenarios. The order in which participants saw prosocial or antisocial trials was randomised.

During each trial, participants were first shown (for 3 s) a sentence and image that depicted either a prosocial or antisocial behaviour (see Fig. 1). Then they rated how likely they would be to engage in that behaviour, using a computer mouse to move a slider on a visual analogue scale (Rating 1; no time restriction). The rating scale was anchored with the words “Never” at its leftmost point and “Always” at its rightmost point. When participants were required to make a rating, the slider first appeared at a random position on the scale in order to avoid any consistent anchoring bias. The position chosen by the participant was recorded to two decimal places (Never = 0.00; Always = 10.00). Next, participants were shown (for 2 s) a screen saying “11-16-year-olds said”, and were then shown a rating of the same scenario, purportedly the average answer provided by a group of 11–16 years olds (this was the age group initially tested as part of the MYRIAD study; data from 17 to 18 year olds were later collected to widen the age range). This provided rating (2 s) was in fact a random number generated between 2 and 8; this range was used instead of 1–10 to ensure the figure was plausible as an average rating. Finally, participants were shown a screen saying, “Please rate again” (2 s), and then were asked to rate again how likely they would be to engage in that behaviour (Rating 2; no time restriction). The whole task took approximately 13 min (see Fig. 1 for trial sequence) and was programmed using Cogent 2000 (Cogent 2000; Team, 2015) and run in MATLAB version R2015a (The Mathworks, 2015) on 13-inch laptops.

Following the end of the study, participants were debriefed and informed that the ratings from other participants were in fact randomly generated.

### 3.3 Statistical analysis

Linear mixed-effects models were used for all analyses. All statistical analyses were conducted in R (R Core Team, 2013) using lme4 (Bates, Mächler, Bolker, & Walker, 2014) and were based on the models used by Foulkes et al. (2018). The categorical variables were coded as: social condition (1 = prosocial, 2 = antisocial), pubertal status (1 = early-mid, 2 = late-post), gender (0 = female, 1 = male) and direction of influence (1 = lower, 2 = higher).

*Hypothesis 1 and 2* *Age differences in susceptibility to social influence and the effect of social condition on susceptibility to social influence.*

This analysis investigated the degree to which participants changed their ratings after seeing the provided rating, and whether the extent of this change depended on age and/or the social condition. Because the provided rating was a randomly generated number between 2.00 and 8.00, it was not related in any systematic way to Rating 1.

The dependent variable in the model was the absolute difference between the participant's Rating 1 and Rating 2 (*change in rating*). Predictor variables in the model were the absolute difference between the provided rating and Rating 1 (*Δrating*); the main effects of age and social condition; two-way interactions between Δrating and age and Δrating and social condition (*prosocial, antisocial*); and a three-way interaction between Δrating, age and social condition were conducted. The variable Δrating was included in the model as a means of assessing whether the difference in magnitude between the participant's Rating 1 and the provided rating influenced the extent to which they changed their ratings. The model used to test Hypotheses 1 and 2 is summarised as follows:

*Change in rating = Δrating + age + social condition + IQ + gender +*

*(Δrating X age) + (Δrating X social condition) +*

*(Δrating X social condition X age)*

Subject-specific and scenario-specific intercepts were included as random effects and IQ and gender were included as covariates. Social condition was Helmert-coded to follow an orthogonal coding scheme. For further discussion about the use of absolute values in the model, see the Supplementary Material.

Hypothesis 3 *Direction of influence.*

This analysis investigated the degree to which participants changed their ratings *in the direction* of the provided rating and how it varied with age and social condition. Two separate models were run, one for prosocial scenarios and one for antisocial scenarios. As with the analysis for Hypothesis 1 and 2, the dependent variable in the models was the absolute difference between the participant's Rating 1 and Rating 2 (*change in rating*). Predictor variables in the models were *direction of influence* (trials when the provided rating was either *higher* or *lower* than the participant's Rating 1; see Knoll et al., 2017); the main effect of age; and a two-way interaction between direction of influence and age. The models used to test Hypothesis 3 are summarised as follows:

*Change in rating (prosocial scenarios only) = direction of influence + age + gender + IQ +*

*(direction X age)*

*Change in rating (antisocial scenarios only) = direction of influence + age + gender + IQ +*

*(direction X age)*

As with the Hypothesis 1 and 2 analysis, subject-specific and scenario-specific intercepts were included as random effects and IQ and gender were included as covariates.

*Hypothesis 4* *Puberty-related differences in susceptibility to social influence.*

For the subset of participants who completed the puberty questionnaire and the social influence task (n = 369), a secondary analysis was conducted that included pubertal status (early/mid, late/post) as a covariate of interest whilst controlling for age. Given the unbalanced gender split and different timings for the onset of puberty, boys and girls were analysed separately. The model used to test Hypothesis 4 is summarised as follows:

*Change in rating = Δrating + pubertal status + social condition + IQ + age +*

*(Δrating X pubertal status) + (Δrating X social condition) +*

*(Δrating X social condition X pubertal status)*

We also ran quadratic and cubic models for model comparisons; the linear model had the smallest Akaike Information Criterion (AIC = 24879) compared to the quadratic model (AIC = 24882; *p* = .427) and the cubic model (AIC = 24886; *p* = .576) and was therefore the best fit.

All analyses are reported with IQ as a covariate (repeated analyses without IQ as a covariate is reported in the Supplementary Material). Planned comparisons were performed to inspect changes in social influence between puberty groups and social condition using the *lsmeans* package (Lenth, 2016).

## 4 Results

### 4.1 Social influence analysis

We ran a linear mixed-effects model to examine the extent to which participants changed their rating from Rating 1 to Rating 2, after seeing the provided rating purportedly from other people. We also examined whether this was influenced by participant age (Hypothesis 1) and/or social condition (prosocial or antisocial; Hypothesis 2).

There was a significant main effect of Δrating (difference between the provided rating and Rating 1; *p* = .001, see Table 2), indicating that participants demonstrated greater changes from Rating 1 to Rating 2 when the disparity between their Rating 1 and the provided rating was greater. There were also significant main effects of age (*p* = .002) and social condition (*p* = .028), suggesting that the difference between Rating 1 and Rating 2 was smaller with increasing age, and smaller for prosocial relative to antisocial scenarios. However, note that we were primarily interested in the *interaction* between Δrating and the other variables. Δrating takes into account the provided rating, and therefore indicates the extent to which social influence has occurred. Assessing interactions between Δrating and the other variables allows us to assess whether the extent of social influence was dependent on age, social condition or both.

**Table 2:** Chi square and parameter estimates (and standard errors) of the main model predicting *change in rating* (absolute difference between Rating 1 and Rating 2) as a function of the main effects (Δrating, age, social condition) and the interactions between the main effects when controlling for IQ and gender.

	χ ^2^	Estimates	SE
Intercept	103.827	1.935***	0.190
Delta rating	10.664	0.203**	0.062
Age	9.348	−0.034**	0.011
Social condition	4.835	0.042*	0.019
Gender	0.033	0.003	0.016
IQ	64.405	−0.007***	0.001
Delta rating x Age	6.988	−0.011**	0.004
Delta rating x Social condition	0.887	−0.026	0.027
Delta rating x Age x Social condition	0.840	0.002	0.002

*Note:* ***p < .001; **p < .01; *p < .05.

**Fig. 2:**
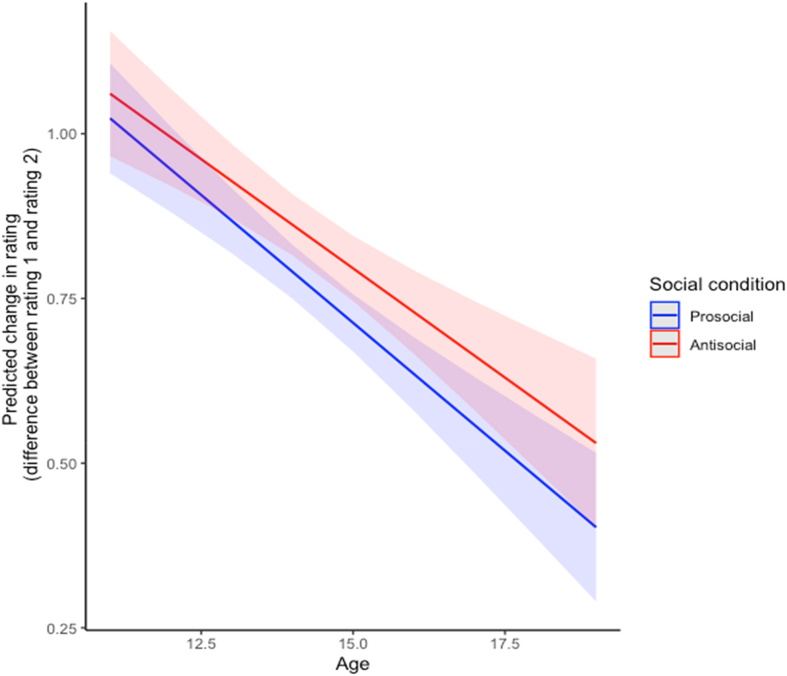
Predicted values for the average change in prosocial and antisocial rating predicted by the difference between the provided rating and the first rating (Δrating), shown across age. The slopes were calculated using estimates of the linear mixed-effect models.

There was a significant interaction between age and Δrating (*p* = .008, see Table 2) on change in ratings, indicating that social influence decreases linearly with age and thus supporting Hypothesis 1 (see Fig. 2). However, there was no interaction between Δrating and social condition on change in ratings (*p* = .350) nor a three-way interaction between Δrating, social condition and age (*p* = .359) either (Hypothesis 2).

### 4.2 Direction of influence analysis

We also ran two linear mixed-effects models (prosocial and antisocial separately) to examine the effect of direction of influence (Hypothesis 3) and whether this was influenced by participant age.

**Fig. 3:**
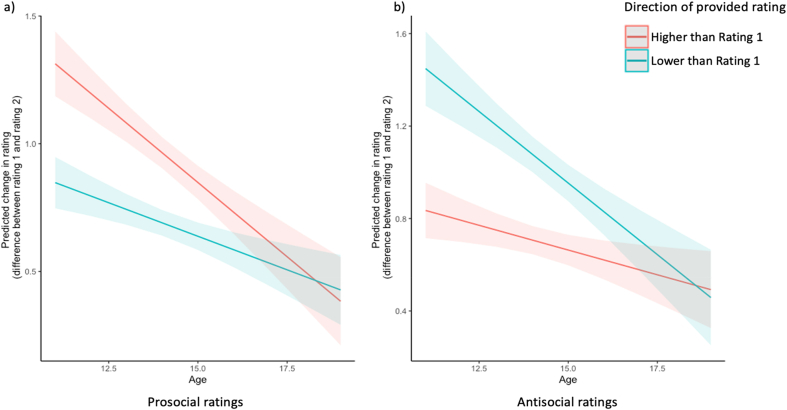
The slopes for the average change in a) prosocial and b) antisocial ratings predicted by direction of influence (provided rating being higher or lower than the participant's Rating 1), shown across age. The slopes were calculated using estimates of the linear mixed-effect model. For a) prosocial ratings, younger adolescents were more influenced when the provided rating was higher than their rating 1 (i.e. more prosocial). For antisocial ratings b) younger adolescents were more influenced when the provided rating was lower than their rating 1 (i.e. less antisocial).

For prosocial scenarios, there was a significant main effect of direction (*p* < .001) and a significant interaction between direction and age on change in rating (*p* = .001, see Table 3, Fig. 3a). This indicated that, when the provided rating was higher than their Rating 1, participants were more likely to change their Rating 2 in line with the provided rating (i.e. change their rating to be more prosocial) than when the provided rating was lower than their Rating 1. This difference in the direction of influence decreased with age.

**Table 3:** Chi square and parameter estimates (and standard errors) of the models (prosocial and antisocial condition separately) predicting *change in rating* (absolute difference between Rating 1 and Rating 2) as a function of the main effects (direction of influence, age) and the interactions between the main effects when controlling for IQ and gender.

	Prosocial			Antisocial		
	χ ^2^	Estimate	SE	χ ^2^	Estimate	SE
Intercept	132.182	3.296***	0.287	57.454	2.191***	0.289
Direction of influence	17.380	−1.166***	0.280	20.140	1.505***	0.335
Age	44.335	−0.116***	0.017	6.742	−0.043**	0.016
Gender	0.899	−0.021	0.022	1.837	0.037	0.027
IQ	30.667	−0.007***	0.001	27.929	−0.008***	0.001
Direction of influence x Age	10.769	0.064**	0.019	12.279	−0.081***	0.023

*Note:* ***p < .001; **p < .01; *p < .05.

For antisocial scenarios, there was also a significant main effect of direction (*p* < .001) and a significant interaction between direction and age on change in rating (*p* < .001, see Table 3, Fig. 3b). This indicated that when the provided rating was lower than their Rating 1, participants were more likely to change their Rating 2 in line with the provided rating (i.e. change their rating to be less antisocial) than when the provided rating was higher than their Rating 1. This difference in the direction of influence decreased with age.

### 4.3 Puberty analysis

To investigate Hypothesis 4 *(puberty-related differences in susceptibility to social influence),* we divided participants according to their pubertal status and gender.

**Fig. 4:**
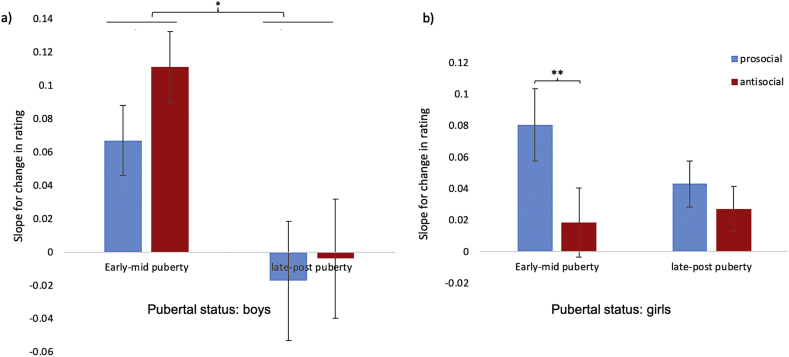
The slope for change in rating by pubertal status for boys (a) and girls (b). The Y-axis shows the slopes for the average change in ratings (difference between rating 1 and rating 2) predicted by the difference between the provided rating and the first rating (Δrating). The slopes were calculated using estimates of the linear mixed-effect models. Error bars represent standard error (**p < .005, *p < .05 Bonferroni-corrected).

For boys*,* the Δrating x puberty interaction was significant (*p* = .006, see Table 4), suggesting that the extent to which male participants changed their ratings was associated with pubertal status, independent of age. There was no significant interaction between Δrating and social condition (*p* = .237) or a three-way interaction between Δrating, social condition and puberty on change in ratings (*p* = .338, see Table 4 and Fig. 4a). Planned comparisons indicated that the early/mid pubertal group were significantly more socially influenced than the late/post pubertal group (*t* (304) = 2.77, *p* = .006; see Fig. 4a).

**Table 4:** Chi square and parameter estimates (and standard errors) of the models (boys and girls separately) predicting *change in rating* (absolute difference between Rating 1 and Rating 2) as a function of the main effects (Δrating, pubertal status, social condition) and the interactions between the main effects when controlling for IQ.

	Boys			Girls		
	χ ^2^	Estimate	SE	χ ^2^	Estimate	SE
Intercept	13.380	1.339***	0.366	94.454	2.328***	0.240
Delta rating	4.788	0.039*	0.018	13.262	0.042***	0.012
Pubertal status	0.360	0.028	0.046	3.876	0.062*	0.032
Social condition	0.001	0.001	0.034	3.898	0.049*	0.025
IQ	8.195	−0.005**	0.002	16.470	−0.005***	0.001
Age	0.557	−0.016	0.021	34.201	−0.082***	0.014
Delta rating x Pubertal status	7.668	−0.050**	0.018	0.376	−0.007	0.012
Delta rating x Social condition	1.401	0.014	0.012	6.229	−0.019*	0.008
Delta rating x Pubertal status x Social condition	0.917	−0.008	0.008	5.591	0.012*	0.005

*Note:* ***p < .001; **p < .01; *p < .05.

For girls, although the Δrating x puberty interaction was not significant (*p* = .540), there was a significant interaction between Δrating and social condition (*p* = .013) and a significant three-way interaction between Δrating, social condition and puberty, on change in ratings (*p* = .018, see Table 4 and Fig. 4b). Planned comparisons indicted that this was driven by the early/mid group being significantly more socially influenced by prosocial than antisocial scenarios (*t* (3401) = 3.02, *p* = .003); see Fig. 4b). All other differences were non-significant (*ps* > .150).

## 5 Discussion

The current study investigated the effects of age and puberty on susceptibility to prosocial and antisocial influence in adolescence. We found that both prosocial and antisocial influence decreased with age between 11 and 18 years, with younger adolescents being more likely to report engaging in prosocial behaviours and less likely to engage in antisocial behaviours after seeing others’ ratings. Antisocial and prosocial influence significantly decreased across pubertal maturation (independent of age) for boys but not girls.

### 5.1 Age and social condition

Our main hypothesis was that social influence would decrease with age *(*Hypothesis 1*: Age differences in susceptibility to social influence).* After providing their first rating (Rating 1), participants were shown ratings purported to be from other adolescents and were then asked to rate the scenario again (Rating 2). Participants showed evidence of social influence in that they changed their second rating more when there was a greater difference between their first rating and the rating they believed came from other adolescents. This is in line with previous studies showing that adolescent participants were socially influenced by others' perceptions of risk (Knoll et al., 2017; 2015) and others’ prosocial behaviour (Foulkes et al., 2018). However, unlike the current study, these previous studies investigated negative outcomes (perception of risky behaviours) and positive outcomes (prosocial behaviour) separately rather than in a single paradigm within the same individuals. Our findings revealed a significant negative linear association between age and the susceptibility to prosocial and anti-social influence. In the study by Foulkes et al. (2018), children (8–11 years), young adolescents (12–14 years) and mid-adolescents (15–18 years) all showed susceptibility to prosocial influence, but prosocial influence did not significantly decrease between these three age groups. One possible explanation for the discrepancy is that the sample size of the adolescent group in the Foulkes et al. (2018) study was almost half that in the present study and thus had lower power. In addition, the testing environments of the two studies were different: the Foulkes et al. study took place in a museum whereas the present study took place in school classrooms. There were differences between study participants and the testing environment: the Foulkes et al. (2018) study consisted of museum visitors who were tested in a quiet room with a small number of strangers, whereas in the present study students were tested in classrooms along with other students. Moreover, the current study also used antisocial scenarios as well as prosocial scenarios, and this may have affected how participants responded. Alternatively, the findings of the current study might be explained by overall changes in the ability to resist peer influence and make more independent decisions in late adolescence (Steinberg & Monahan, 2007; Sumter et al., 2009).

Our findings also support previous studies which have shown that susceptibility to antisocial influence decreases with age across adolescence. The majority of previous studies have focussed on direct forms of antisocial behaviour, for example, greater exposure to antisocial peers increases adolescent offending behaviours (Tatar, Cavanagh, & Cauffman, 2016) and bullying (Doehne et al., 2018), with few studies investigating indirect antisocial behaviour such as ostracising others (Sijtsema et al., 2014). Even though the social influence effects in the present study pertain specifically to changes in hypothetical antisocial actions, and not actual behaviours, our findings support these previous findings by showing that antisocial influence decreases with age.

### 5.2 Direction of influence

Another aim of the current study was to understand whether social influence would be affected by the *direction* of other adolescents' ratings and whether this differs across social condition and age (Hypothesis 3*: direction of influence*). We found that social influence was affected by lower and higher ratings and such influence decreased with age. Specifically, younger participants were more socially influenced when the prosocial provided rating was higher than their initial rating (i.e. more prosocial) and when the antisocial provided rating was lower than their initial rating (i.e. less antisocial) (see Fig. 3), in line with our hypothesis. In Knoll et al.’s (2017) risk perception paradigm, younger participants were more strongly influenced by ratings provided by teenagers than by adults, but only when the teenage provided rating was more risky than the participant's own rating. Whilst this measure of risk perception is different to the prosocial and antisocial measures in the current study, both studies demonstrate that younger adolescents are more easily influenced by other adolescents who report being more risk averse or more prosocial than the participant. Our findings suggest that adolescents are more likely to report engaging in prosocial behaviours and less likely to engage in antisocial behaviours after seeing ratings of other adolescents, and that this social influence declines with age, possibly reflecting genuine socialisation effects as opposed to arbitrary changes in ratings. We speculate that this is at least partly because younger adolescents are still trying to come to terms with larger school contexts and “fitting in” by using positive impression management (Fine, 2004; McElhaney, Antonishak, & Allen, 2008).

### 5.3 Dissociable effects of age and puberty

Previous work investigating social influence has primarily focused on changes across chronological age. However, puberty has been shown to have dissociable effects from age on social-affective development (Bramen et al., 2012; Crone & Dahl, 2012; Forbes et al., 2010; Goddings et al., 2012; Wierenga et al., 2018). In the present study, we measured pubertal status to assess puberty-related differences in susceptibility to social influence *(*Hypothesis 4*)* in boys and girls separately. Our hypotheses were speculative as there is no previous research on susceptibility to social influence and pubertal status. Our results showed that boys in early/mid puberty were more socially influenced than boys in the late/post pubertal group on both antisocial and prosocial scenarios, independent of age. In contrast, early/mid pubertal girls were more influenced by prosocial than antisocial scenarios, independent of age. This suggests that the extent to which boys changed their ratings was affected only by pubertal status (regardless of social condition), whereas for girls the extent of social influence depended on both pubertal status and social condition. It is not clear why the three-way interaction between Δrating, social condition and puberty was only seen in girls and not in boys. Further research is required to examine the relationship between gender, pubertal development, and type of social influence.

The finding that social influence did not significantly decrease with increasing pubertal maturity for girls is in line with studies suggesting that girls are more susceptible to implicit social influence (Hanish, Martin, Fabes, Leonard, & Herzog, 2005; Iscoe, Williams, & Harvey, 1963). In the present study, the provided ratings can be considered as implicit social influence as they were from unknown adolescents and referred to hypothetical actions. This could have a stronger influence effect on girls as boys appear to be more affected by explicit and overt attempts of pressure from their peers (Berndt, 1979; McCoy, Dimler, Samuels, & Natsuaki, 2019; Rose & Rudolph, 2006).

### 5.4 Limitations

A number of limitations of the present study need to be mentioned. With morally relevant behaviour such as prosocial behaviour, there is some evidence that what people report they will do and what they actually do differs (e.g., Teper, Inzlicht, & Page-Gould, 2011). For example, children and adolescents say they will give more than the amount they actually give in Dictator games (Blake, 2018). Young people may boast about engaging in antisocial behaviours as they may be considered as status enhancing (Sijtsema, Garofalo, Jansen, & Klimstra, 2019). Furthermore, the source of influence in the present study were unfamiliar adolescents. However, studies have shown that peer acceptance and friendship quality affect how readily adolescents conform to their friends’ behaviours: Urberg, Luo, Pilgrim, and Degirmencioglu (2003) found that, for cigarette and alcohol use, adolescents who reported high levels of positive quality in their closest friendship were more influenced by that relationship than were those whose relationships were less positive. It would be interesting for future studies to address the questions asked in the current study using real ratings from close peers.

Due to time constraints, only 16 out of the possible 82 scenarios were administered during the task and therefore the extent to which adolescents changed their ratings may have been impacted by the topic of the randomly selected scenarios that they saw. We attempted to control for this issue by including subject-specific and scenario-specific intercepts as random effects in the model so that general effects relating to specific scenarios are accounted for (in line with the analyses of our previous studies (Foulkes et al., 2018; Knoll et al., 2017; 2015)). The limited number of scenarios and two specified random effects may have underpowered the linear mixed-effects models and potentially biased the parameter estimates. We ran a new model that did not include these random effects and ran a model comparison with the original model (including random effects). We found that the original model with the two random effects had a significantly smaller Akaike Information Criterion (AIC = 24879) compared to the model without any random effects (AIC = 25980; *p* < .001) and was therefore the best fit. Nevertheless, future studies should aim to include a larger number of scenarios.

Future studies could also include a larger range of prepubertal to post-pubertal participants as well as measures of environmental factors, such as position within the peer group, friendship quality, and degree of autonomy. This would clarify whether the heightened susceptibility to peer influence in adolescence is the result of biological (hormonal) processes associated with puberty or social factors associated with advanced puberty and increasing age. Longitudinal designs would enable these relationships to be assessed over time.

### 5.5 Implications

Understanding what makes some adolescents more susceptible than others to peer influence is important for the design of effective prevention programs aimed at reducing antisocial behaviour and promoting prosocial behaviour. Indeed, studies that target social norms through peer-led interventions have shown positive outcomes across a number of domains such as bullying (Paluck, Shepherd, & Aronow, 2016) and smoking (Campbell et al., 2008). In one study, 56 middle schools in the USA (with children aged 11–16 years) were randomly allocated to either a peer led anti-bullying programme or practise as usual (Paluck et al., 2016). In this programme, a number of students who had a large number of positive social connections among their peers (socially referent students) attended an anti-conflict programme and were encouraged to lead grassroots anti-bullying campaigns within their schools. Compared with control schools, the schools in which the anti-bullying programmes were led by students saw a 25% reduction in conflict over the ensuing year. The effect was strongest in schools with a higher proportion of socially referent students leading the campaigns. The study demonstrated the power of peer influence in changing behaviour in adolescents. The findings of the current study suggest that targeting young, early pubertal adolescents may be even more effective.

## 6 Conclusion

The present study investigated susceptibility to prosocial and antisocial influence across adolescence and found that both types of social influence decreased with age, with younger adolescents being more likely to report engaging in prosocial behaviours and less likely to engage in antisocial behaviours after seeing others’ ratings. Pubertal maturation was independently associated with a decrease in social influence in boys but not girls. Overall the findings demonstrate the relationship between social influence and maturity is dependent on the nature of the social influence (positive versus negative) and on gender. The current findings highlight the importance of measuring puberty as well as age when understanding decision-making and changes in social cognition across adolescence.

## Declaration of competing interest

None.
