# Supplementary material for: Susceptibility to prosocial and antisocial influence in adolescence
Source: J Adolesc. 2020 Oct;84:56–68. doi: 10.1016/j.adolescence.2020.07.012 (PMC7674583; doi:10.1016/j.adolescence.2020.07.012)
Supplement: Multimedia component 1 [file mmc1.docx]

# **Supplementary Materials**

## **Method**

*Full list of prosocial scenarios*

Visit a friend when they are ill

Care for a friend when they are ill

Give up your seat for a friend on the bus

Give up your seat for a family member on the bus

Give up your seat to a stranger on the bus

Give up your seat for a friend on the train

Give up your seat to a stranger on the train

Carry a friend's bag for them

Carry a family member's bag for them

Make a friend a birthday card

Make a family member a birthday card

Buy a friend a birthday card

Buy a family member a birthday card

Stand up for a classmate when they are being teased

Defend a classmate when they are being bullied

Give something you like to charity

Lend a friend your favourite book

Let a friend go ahead of you in a queue

Let a classmate go ahead of you in a queue

Lend a friend your favourite clothes

Raise money for charity

Buy a friend a birthday present

Give money to charity

Show a stranger where to go if they are lost

Make a friend a present

Make a family member a present

Offer to help around the house

Lend a friend money

Lend a family member money

Volunteer for a charity

Help a stranger if they have fallen

Help a friend with their schoolwork

Help a classmate with their schoolwork

Share your revision notes with a friend

Share your revision notes with a classmate

Like a friend's post on Facebook

Compliment a family member

Message a friend to see how they are

Sponsor a friend for charity

Sponsor a classmate for charity

Sponsor a family member for charity

*Full list of antisocial scenarios*

Shout at a family member in an argument

Shout at a friend in an argument

Take something that doesn’t belong to you

Laugh at a friend when they make a mistake

Laugh at a classmate when they make a mistake

Laugh at a family member when they make a mistake

Look through a friend's phone without asking

Look through a classmate's phone without asking

Look through a family member's phone without asking

Push in front of friend in a queue

Gossip about a classmate

Gossip about a friend

Gossip about a friend online

Talk about a friend behind their back

Talk about a classmate behind their back

Laugh at someone's clothes

Laugh at someone's work

Look through a family member's room when they are away

Make a mess at home and not clear it up

Ignore a friend online

Ignore a friend's Whatsapp messages

Make fun of a friend

Make fun of a classmate

Tease a friend

Tease a family member

Tease a classmate

Tell someone's secret

Trip up a classmate as a joke

Trip up a friend as a joke

Hit a friend when you are angry

Lie to a teacher to get out of trouble

Lie to a parent to get out of trouble

Write or draw on a desk at school

Answer back to a teacher in a rude way

Pretend you are someone else online

Ignore what a teacher asks you to do

Blame a friend for something you did wrong

Blame a classmate for something you did wrong

Blame a family member for something you did wrong

Swear at a friend in an argument

Swear at a family member in an argument

**Use of absolute values in main models**

Note that, as in previous studies that used similar analyses (e.g. Foulkes et al. 2018; Knoll et al. 2015), absolute values of *change in rating* and *Δrating* were used in the model, rather than positive and negative values. This was because positive and negative values of *change in rating* both represent social influence, depending on the trial. It was not the case that a positive value would represent *more* social influence than a negative one. In other words, it was meaningful that a participant had changed their rating by e.g. five absolute points between their first and second rating, but not meaningful (in this paper) whether this was because the participant had been influenced to increase or decrease their first answer. One paper specifically addresses whether the direction of influence is relevant in social influence (Knoll et al., 2017), but in this paper we were interested simply in the magnitude of social influence.

However, a potential issue with using absolute values arises when considering Δrating. This is that a trial in which a participant sees the provided rating and then changes their answer in the *opposing* direction to the provided rating will be treated in the model in the same way as a trial in which the participant changes their answer to be *closer* to the provided rating. For example, a participant who first rates 5, sees a provided rating of 8, and then increases their answer to 7 will receive the same absolute Δrating (i.e. 2) as a participant who first rates 5, sees provided rating of 8, and then drops their answer to 3. This is a potential problem because only the former scenario should be considered social influence, but both are treated as social influence in the model. One possible solution was to include rating 1 in the model in order to provide an ‘anchor’ for Δrating, but this led to multicollinearity as rating 1 was in the model as a predictor twice (once on its own and once as part of Δrating). However, there were only a minority (6.33%) of trials in which participants rated substantially in the opposing direction (greater than 2 points). Therefore, the original model with absolute values was kept, and all trials were retained in the analysis.

## **Results**

All analyses without controlling for IQ

We conducted the same analyses for hypothesis 1 (Age differences in susceptibility to social influence) and hypothesis 2 (Effect of social condition) but without controlling for IQ. The model was otherwise the same as the main social influence analysis, and can be represented as follows:

*Change in rating = Δrating + age + social condition + gender +*

*(Δrating X age) + (Δrating X social condition) +*

*(Δrating X social condition X age) + (1|subject/scenario)*

Supplementary Table 2. Chi square and parameter estimates (and standard errors) of the main model predicting *change in rating* (absolute difference between Rating 1 and Rating 2) as a function of the main effects (Δrating, age, social condition) and the interactions between the main effects when controlling for gender.

|  | **χ ^2^** | **Estimates** | **SE** |
| --- | --- | --- | --- |
| Intercept | 48.866 | 1.120*** | 0.160 |
| Delta rating | 8.842 | 0.193** | 0.065 |
| Age | 8.622 | -0.033** | 0.011 |
| Social condition | 5.487 | 0.044* | 0.019 |
| Gender | 0.274 | 0.009 | 0.016 |
| Delta rating x Age | 5.516 | -0.011* | 0.004 |
| Delta rating x Social condition | 0.848 | -0.025 | 0.027 |
| Delta rating x Age x Social condition | 0.689 | 0.002 | 0.002 |
| *Note:* *** *p*<.001; ** *p*<.01; * *p*<.05 |  |  |  |
|  |  |  |  |

There was a significant main effect of Δrating, indicating that participants demonstrated greater changes from Rating 1 to Rating 2 when the disparity between their Rating 1 and the provided rating was greater (see Table 2).

There was a significant interaction between age and Δrating on change in ratings, indicating that social influence decrease linearly with age and therefore supporting hypothesis 1. However, there was no interaction between Δrating and social condition on change in ratings or a three-way interaction between Δrating, age and social condition (hypothesis 2), in line with the analysis controlling for IQ.

We conducted the same analyses for hypothesis 3 (direction of influence) without controlling for IQ. The model was otherwise the same as the main analysis, and is shown below:

*Change in rating = direction of influence + age + gender +*

*(direction of influence x age) + (1|subject/scenario)*

Supplementary Table 3. Chi square and parameter estimates (and standard errors) of the models (prosocial and antisocial condition separately) predicting *change in rating* (absolute difference between Rating 1 and Rating 2) as a function of the main effects (direction of influence, age) and the interactions between the main effects when controlling for gender.

|  |  | **Prosocial** | | |  | **Antisocial** | | |
| --- | --- | --- | --- | --- | --- | --- | --- | --- |
|  |  |  |  |  |  |  |  |  |
|  |  | **χ ^2^** | **Estimate** | **SE** |  | **χ ^2^** | **Estimate** | **SE** |
| Intercept |  | 81.718 | 2.446*** | 0.271 |  | 27.388 | 1.263*** | 0.241 |
| Direction of influence |  | 16.516 | -1.144*** | 0.281 |  | 19.924 | 1.495*** | 0.335 |
| Age |  | 32.573 | -0.107*** | 0.019 |  | 5.318 | -0.039* | 0.017 |
| Gender |  | 0.321 | -0.015 | 0.026 |  | 1.277 | 0.032 | 0.028 |
| Direction of influence x Age |  | 9.984 | 0.062** | 0.020 |  | 11.956 | -0.080*** | 0.023 |
| *Note:* *** *p*<.001; ** *p*<.01; * *p*<.05 |  |  |  |  |  |  |  |  |

For prosocial scenarios, there was a significant main effect of direction and a significant interaction between direction and age on change in rating (see Table 3). This indicated that, when the provided rating was higher than their rating 1, participants were more likely to change their rating 2 in line with the provided rating and this difference in the direction of influence decreased with age.

For antisocial scenarios, there was also significant main effect of direction and a significant interaction between direction and age on change in rating (see Table 3). This indicated that when the provided rating was lower than their rating 1, participants were more likely to change their rating 2 in line with the provided rating and this difference in the direction of influence decreased with age. This is in line with the main analyses.

We conducted the same analyses for hypothesis 4 (Pubertal differences in susceptibility to social influence) but without controlling for IQ. The model was otherwise the same as the main social influence analysis, and can be represented as follows:

*Change in rating = Δrating + puberty + social condition + age + gender +*

*(Δrating X puberty) + (Δrating X social condition) +*

*(Δrating X social condition X puberty) + (1|subject/scenario)*

Supplementary Table 4. Chi square and parameter estimates (and standard errors) of the models (males and females separately) predicting *change in rating* (absolute difference between Rating 1 and Rating 2) as a function of the main effects (Δrating, pubertal status, social condition) and the interactions between the main effects.

|  |  | **Males** | | |  | **Females** | | |
| --- | --- | --- | --- | --- | --- | --- | --- | --- |
|  |  |  |  |  |  |  |  |  |
|  |  | **χ ^2^** | **Estimate** | **SE** |  | **χ ^2^** | **Estimate** | **SE** |
| Intercept |  | 5.968 | 0.751* | 0.307 |  | 79.583 | 1.757*** | 0.197 |
| Delta rating |  | 4.709 | 0.040* | 0.018 |  | 12.862 | 0.042*** | 0.012 |
| Pubertal status |  | 0.250 | 0.023 | 0.046 |  | 3.833 | 0.062 | 0.032 |
| Social condition |  | 0.005 | 0.002 | 0.034 |  | 4.037 | 0.050* | 0.025 |
| Age |  | 0.360 | -0.013 | 0.021 |  | 33.300 | -0.081*** | 0.014 |
| Delta rating x Pubertal status |  | 7.323 | -0.050** | 0.018 |  | 0.363 | -0.007 | 0.012 |
| Delta rating x Social condition |  | 1.359 | 0.014 | 0.012 |  | 6.523 | -0.020* | 0.008 |
| Delta rating x Pubertal status x Social condition |  | 0.902 | -0.008 | 0.008 |  | 5.779 | 0.012* | 0.005 |
| *Note:* *** *p*<.001; ** *p*<.01; * *p*<.05 |  |  |  |  |  |  |  |  |

For boys, the Δrating x puberty interaction was significant, suggesting that the extent to which boys were socially influenced was affected by pubertal status, independent of age (see Table 4), in line with the analysis controlling for IQ. There was no significant interaction between Δrating and social condition or a three-way interaction between Δrating, social condition and puberty on change in ratings, also in line with the analysis controlling for IQ. Planned comparisons indicated that the early/mid pubertal group were significantly more socially influenced than the late/post pubertal group (*p*=.014).

For girls, although the Δrating x puberty interaction was not significant, there was a significant interaction between Δrating and social condition and a significant three-way interaction between Δrating, social condition and puberty, on change in ratings (see Table 4). Planned comparisons indicted that this was driven by a significant difference between antisocial and prosocial scenarios within the early/mid female group (*p*=.003).
